# Computational Spectroscopy in Solution by Integration of Variational and Perturbative Approaches on Top of Clusterized Molecular Dynamics

**DOI:** 10.1021/acs.jctc.0c00454

**Published:** 2020-07-22

**Authors:** Giordano Mancini, Sara Del Galdo, Balasubramanian Chandramouli, Marco Pagliai, Vincenzo Barone

**Affiliations:** † Scuola Normale Superiore di Pisa, Piazza dei Cavalieri 7, I-56126 Pisa, Italy; ‡ Istituto Nazionale di Fisica Nucleare (INFN) sezione di Pisa, Largo Bruno Pontecorvo 3, 56127 Pisa, Italy; § Super Computing Applications and Innovation, CINECA, Via Magnanelli, 6/3, 40033 Casalecchio di Reno, BO, Italy; ∥ Dipartimento di Chimica “Ugo Schiff”, Università degli Studi di Firenze, Via della Lastruccia 3, 50019 Sesto Fiorentino, Italy

## Abstract

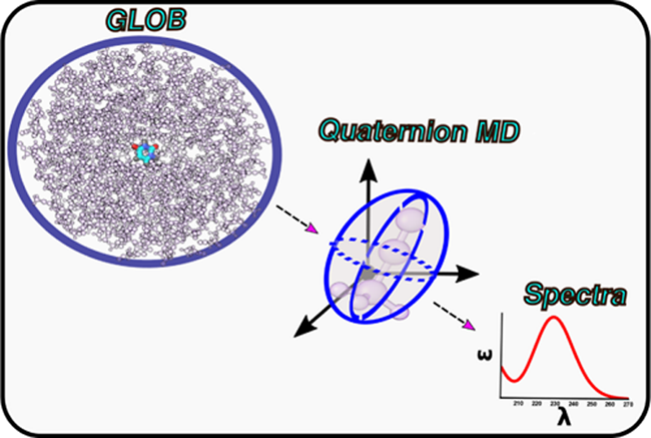

Multiscale QM/MM approaches have become the most suitable and effective methods for the investigation of spectroscopic properties of medium- or large-size chromophores in condensed phases. On these grounds, we are developing a novel workflow aimed at improving the generality, reliability, and ease of use of the available tools. In the present paper, we report the latest developments of such an approach with specific reference to a general workplan starting with the addition of acetonitrile to the panel of solvents already available in the General Liquid Optimized Boundary (GLOB) model enforcing nonperiodic boundary conditions (NPBC). Next, the solvatochromic shifts induced by acetonitrile on both rigid (uracil and thymine) and flexible (thyrosine) chromophores have been studied introducing in our software a number of new features ranging from rigid-geometry NPBC molecular dynamics based on the quaternion formalism to a full integration of variational (ONIOM) and perturbative (perturbed matrix method (PMM)) approaches for describing different solute–solvent topologies and local fluctuations, respectively. Finally, thymine and uracil have been studied also in methanol to point out the generality of the computational strategy. While further developments are surely needed, the strengths of our integrated approach even in its present version are demonstrated by the accuracy of the results obtained by an unsupervised approach and coupled to a computational cost strongly reduced with respect to that of conventional QM/MM models without any appreciable accuracy deterioration.

## Introduction

Prediction of the spectra of medium-size semirigid chromophores in the gas phase is a nontrivial problem, needing a careful balance between feasibility and accuracy.^1^ The study of flexible molecules in condensed phases is further complicated by the necessity of an exhaustive sampling of both internal soft degrees of freedom and environmental effects.^2−5^ As widely recognized, both the quality of the sampling and the accuracy of the quantum mechanical model concur to shape the computed spectra, not to speak about the ill-defined role of possible error compensations.

The most effective solution to this problem is offered by multiscale strategies like quantum mechanics–molecular mechanics (QM/MM) approaches in which a relatively small part of the system (e.g., the chromophore) is treated at the highest possible QM level, whereas the remaining (huge) part (including remote regions of the solute and the solvent possibly beyond the cybotactic zone) is treated at a lower QM or MM level.^6−10^

When dealing with complex systems, the whole route from the design of the study to its final accomplishment involves the clever management of a number of tricky aspects. Therefore, only well-devised and purposely tailored strategies can provide a satisfactory modeling, since each step of the overall procedure requires a fine tuning of the accuracy/cost ratio, which must be balanced with that of the other steps and with the final sought accuracy. In this framework, the main aim of this contribution is to present some of the latest developments we have implemented into a general workflow for the study of the spectroscopic features of medium-to-large-size chromophores in condensed phases. This effort is based on our opinion that computational spectroscopy will not become a routine companion of experimental studies in the analysis of challenging systems until general and user-friendly tools have been developed and validated.^11^ Broadly speaking, a general QM/MM tool includes three main ingredients: (i) classical sampling of the complete system; (ii) selection of a representative number of system configurations for performing the successive high-level calculations, and (iii) QM/MM calculations for the chosen structures. Even if the attention is often focused only on the last topic, all of the ingredients often play a comparable role in determining the final accuracy of the results.

The description of a molecular system at the MM level requires a set of parameters encoding its properties (force field (FF)). Since the accuracy of the classical sampling strictly depends on the quality of the force-field (FF) parameters, the availability of an accurate FF is the mandatory first step of any successful modeling.^10,12,13^

Once an FF is available, molecular dynamics (MD) simulations can be used to sample the phase space usually employing periodic boundary conditions (PBC).^14^ Unfortunately, PBC are not free from possible artifacts for intrinsically nonperiodic systems,^15^ and therefore, several alternative strategies enforcing nonperiodic boundary conditions (NPBC) have been proposed.^16,17^ According to this general paradigm, a finite system (generally a sphere) containing the solute and a sufficient number of explicit solvent molecules is embedded within a polarizable continuum mimicking bulk solvent effects, thus avoiding spurious anisotropic solvation effects and periodicity artifacts. Besides, the computational cost is significantly reduced due to the lower number of explicit molecules required to fill the sphere compared to other cell structures (e.g., cubic box). One of such mixed discrete/continuum approaches is the so-called general liquid optimized boundary (GLOB) model,^18^ which relies on a mean-field-based approach to account for the interaction with the continuum. GLOB has been applied to study various systems and properties in aqueous solution,^19−21^ and recently, to model also nonaqueous media.^22,23^ Further, the applicability of the model has been tested for scenarios where the use of polarizable force fields is of particular relevance.^23^ In recent contributions,^22−24^ we have presented a novel MD engine embedded in the Gaussian suite of programs and working within the GLOB paradigm. The code has been used to perform MD simulations using both fixed and fluctuating charges^25^ (FQ) in aqueous or organic solvents using fully flexible models or constrain-based methods such as SHAKE^26^ or SETTLE.^27^ However, for relatively small (but more complex than trigonal cases) solvent molecules, a rigid-body (RB) representation would improve the stability and accuracy of the simulation. Therefore, in the present contribution, we have introduced an RB MD integrator (based on quaternions)^28^ and tested its performance within GLOB for nontrigonal molecules (since most studies on quaternion-based dynamics compare it to SHAKE, using water molecules). Once a sufficient MD sampling is achieved, a number of representative configurations of the complete system are extracted for the following QM calculations.

Within the QM/MM paradigm, different schemes have been developed for the treatment of interactions between QM and MM regions. The most refined and widely employed approach is based on the electrostatic embedding (EE) model^4,29^ in which the partial charges of the MM region are included into the QM Hamiltonian through an electrostatic term. This approach includes the polarization of the QM wavefunction by the MM region charges and avoids the approximation of describing the QM fragment in terms of point charges. For this kind of QM/MM calculations, an effective selection of a reduced sampling able to cover most of the system configuration space with the minimum number of snapshots is of paramount relevance to limit the computational effort, which scales with (i) the number of the degrees of freedom of the system and (ii) the sensitivity to conformational fluctuations of the phenomenon under investigation. As a consequence, well-converged simulations of electronic spectra usually require hundred to thousand snapshots distributed over the whole configuration space. The simplest way to perform this subsampling is to extract snapshots from the MM trajectories with a constant step, but this strategy is both inefficient and scarcely insightful. Unsupervised learning (UL) techniques such as clustering,^10,30,31^ self-organizing maps,^32^ and combinatorial optimization^33^ may yield a balanced and efficient subsampling of MM trajectories once an exhaustive overall sampling has been carried out. The application of UL requires the choice not only of an efficient sampling/classification method but also of suitable molecular descriptors for the comparison of structures. These descriptors may be structural properties of the QM fragment (e.g., the orientation of groups on selected rotating bonds) or of the system (e.g. the number of hydrogen bonds between the QM and MM fragments) or electric properties (e.g., the electric field exerted by the MM atomistic environment over the QM fragment) and may be used in combination.

Within the QM/MM framework, the perturbed matrix method (PMM) represents an effective alternative.^34−36^ Contrary to the variational approaches outlined above, the embedding effects exerted by the MM environment on the QM center are treated by a perturbative approach. The core of the method is the diagonalization of the perturbed Hamiltonian matrix expressed in terms of the Hamiltonian eigenstates computed in the absence of the perturbation. The QM computations are carried out for the corresponding fragment in vacuum, while MM simulations of the complete system are exploited to take environmental effects into account. The method has been implemented in a local development version of the Gaussian suite of programs,^37^ and it has also been expanded to include different levels of theory for the treatment of the perturbation term.^38^

Variational and perturbative approaches have been recently combined in the ONIOM/EE-PMM method.^39^ In this approach, a preliminary analysis of the MM sampling is performed to identify a set of clusters or basins for partitioning the trajectory. Then, the ONIOM/EE method is applied only for “reference” snapshots of the simulation representative of each subtrajectory within a single basin. This step allows us to avoid the main potential shortcoming of the PMM, namely, the use of a perturbative approach to describe the (possibly) strong modifications induced by average solvent effects on gas-phase structures and/or spectral features. Next, the PMM is employed to treat local deviations within each cluster, i.e., to model the electrostatic potential fluctuations with respect to the reference configuration. Therefore, a key aspect of the approach is the effective yet reliable definition of the basins, which, for simple systems, can be based on intuitive “visual inspection”. For more complex cases, several automatic clustering procedures have been proposed,^30,31,40^ usually based on the root-mean-square deviation (RMSD) of (nonhydrogen) atoms after a roto-translational fit as a measure of the distance between simulation frames. However, for the accurate simulation of spectroscopic parameters of medium-size chromophores, internal coordinates are more effective. On these grounds, we propose a pipeline joining accuracy, ease of benchmarking (validation is an important but often overlooked aspect of UL applications), and use of the most effective generalized internal coordinates.

The effectiveness of the ONIOM/PMM approach for the description of optical and chiroptical spectra has been recently documented.^41,42^ On these grounds, the main purpose of the present study is to assess the application of RB solvent models under NPBC to the study of organic chromophores in conjunction with the ONIOM/EE-PMM method. Concerning solvents, the already available data for methanol^24^ were refitted to a polynomial form, whereas new simulations of acetonitrile (CH_3_CN) nanodroplets were performed employing a new very reliable force field^43^ to test the RB integrator and to obtain an mean field (FF) component (vide infra for details). Next, we selected two semirigid chromophores, thymine and uracil, whose relatively soft ring deformations can be more effectively described, if needed, by vibrational modulation effects obtained from a harmonic treatment of Franck–Condon and, possibly, Herzberg–Teller contributions.^41^ Finally, we considered a flexible chromophore, tyrosine, whose soft (torsional) degrees of freedom can be well accounted for in the framework of a classical treatment.

The manuscript is organized as follows: in the Methods section, we (i) illustrate how RB algorithms have been integrated in the NPBC framework, (ii) summarize the (MF) optimization, (iii) describe the latest implementations of the ONIOM/EE-PMM approach, and (iv) outline the procedures used to select quantum centers (QCs, which will be explicitly defined in the following); then, computational details and simulation parameters are given. In the section devoted to results, we first analyze the stability of the RB propagation for pure acetonitrile in terms of energy and temperature fluctuations and then proceed to optimize the MF potential of pure solvents by means of long NPBC simulations. Finally, the absorption spectra of the different chromophores in methanol and/or acetonitrile are analyzed, starting from rigid species and then considering flexible ones.

## Methods

### Rigid-Body Dynamics

Under an RB representation, the motion of a molecule is factorized into a translational part, describing the motion of the molecular center of mass (COM) in the laboratory reference frame (LF hereafter) and a rotational part for the RB rotations around the principal inertia axes. Since the geometry is fixed, the latter term can be represented in a fixed reference frame (molecular frame or MF hereafter) so that the molecular rotational motion is just described by the rotations of MF with respect to LF. While the description of the translational part is straightforward and coincides with the dynamics of a particle having the mass of the molecule, rotation can be represented in different ways. The widely used Euler angles lead to singularities and loss of degrees of freedom (misleadingly named “gimbal lock”). On the other hand, quaternions provide a representation that is singularity-free and computationally convenient. For this reason, they are being increasingly used to represent RB orientation in fields such as engineering and computer graphics.^44^ Their use in MD was first proposed by Evans^45,46^ and later by Fincham,^47^ Svanberg,^48^ Omelyan,^49^ and Rozmanov et al.^50^ An exhaustive review of the applications of quaternions in molecular modeling has been given by Karney.^51^ Here, we adapted the rotational velocity Verlet (hereafter RVV1) integrator based on a quaternion representation of rotational motion proposed by Rozmanov et al. (see ref (50)). For the sake of brevity, here, we simply recall the fundamental equation that allows us to rotate a vector  ; a summary of quaternion definition and properties is given in Appendix. A unit quaternion *Q* conveys all of the information about a molecule orientation either in the MF or LF, and the rotation of a vector **v** between reference systems can be achieved with
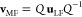
1Backward rotation takes place exchanging *Q* with its inverse *Q*^–1^ = Q*. The rotational motion of a generic molecule *i* is ruled by the inertia tensor **I**_*i*_, the orientation Ω_*i*_, the angular momentum **L**_*i*_, and the torque **T**_*i*_, which are the rotational analogues of mass, position, momentum (or velocity), and force used for rectilinear motions. In particular, the orientation is a function of the angular momentum and inertia tensor and its actual definition depends on how rotational motion is described. The corresponding equations of motion are
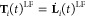
2
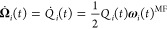
3where ω = **I**_*i*_^–1^(*t*)**L**_*i*_(*t*) is the angular velocity; note that in eq 3 the time derivative of the quaternion at *t* depends on *Q*(*t*) itself. This is best explained following the different steps of the RVV1 algorithm. The initial state for the *i*th molecule is specified by the starting orientation *Q*(0) and COM position **x**_COM_(0), the starting angular momentum **L**^LF^(0) and COM velocity **v**_COM_(0), and the starting total force acting on the COM and torque, **F**_COM_(0) and **T**^LF^(0), respectively. The quantities **x**_COM_(0), **v**_COM_(0), and **F**_COM_(0) are used to describe the translational motion with the standard velocity Verlet (VV) algorithm,^14^ while rotational quantities are used as follows (dropping the index *i*):

1. Laboratory frame quantities are rotated into the molecular frame reference system (LF → MF)
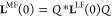
4
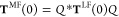
5
2. The angular momentum in molecular frame is updated at  using Euler’s equation
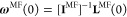
6
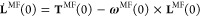
7
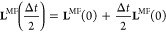
8The angular momentum in the laboratory frame is also updated (using the torque), in analogy with velocity Verlet for a rectilinear motion
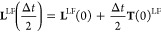
9
3. At this point, the analogues of force and velocity for particle dynamics are updated at ; we need to estimate the orientation *Q*(Δ*t*), which means solving eq 3. The initial estimates of  and  are obtained using eq 3 and then using the half-step quaternion derivative
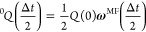
10
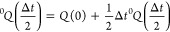
11
4. At this point, we need to evaluate the quaternion derivatives
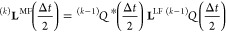
12
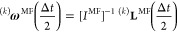
13
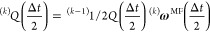
14
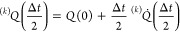
15Once the difference |^(*k*)^*Q* – ^(*k*–1)^*Q*| is less than some threshold ϵ, the system has converged and the final value of *Q*(*t*) can be calculated
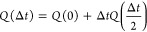
16At this point, the norm of *Q* is calculated; if the deviation from a unit quaternion is small, normalization is enforced applying *Q* = *Q*/|*Q*|, otherwise an error flag is set to signal an unstable system (in our test, this happened only during bug fix)
5. The solution of eq 16 allows the computation of the new orientation for molecule *i* and, together with the COM motion, the calculation of the new position of all atoms in the laboratory frame. The first half-step of RVV1 is now solved, and the new forces and torques at time Δ*t* can be computed.
6. In analogy with VV second half-step, the angular momentum in the laboratory frame is now updated
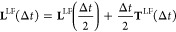
17
7. At this point, a full integration step has been carried out and the simulation can proceed to time (3/2)Δ*t*.

At the beginning of a simulation, the inertia tensor **I** of all rigid fragments is calculated and diagonalized; the rotation matrix that aligns a molecule *i* with its principal axes is converted to a quaternion and used to define its starting orientation in MF, while the diagonal components of **I** are stored. Starting (COM) velocities and angular momenta are assigned by sampling for each fragment from a Gaussian distribution and then scaling the values to obtain the desired kinetic energy. In previous contributions using the GLOB model, the integration time step was limited to 2.0 fs and the solvent used (with the exception of CH_3_CN in ref (24)) enclosed in a sphere. To assure that solvent molecules remained enclosed in the simulation box, an elastic boundary (acting on atomic velocities) was enforced around it; this had the advantage of conserving forces and did not create nonphysical instabilities in the total kinetic energy, which can arise when using a simple repulsive wall with a large time step. However, this approach is straightforward when using nonrigid molecules, but becomes cumbersome when using an RB representation. For this reason, to enforce confinement in the box while keeping spurious boundary effect and computational effort under control, we also added a rough-wall^14^ representation of the boundary for NVT simulations. Whenever a rigid fragment steps beyond the boundary, it is assigned a new random angular momentum and COM velocity (under the constraint that the new velocity cannot be tangential to the boundary or directed outward) sampled at the reference (or current) temperature. If, for any reason, some rigid fragments must be oriented in a predetermined way (not in the present case), the rotation least-square fit method described by Karney^51^ has been implemented.

### Optimization of NPBC Mean Field

A detailed description of the GLOB model is already available in the literature.^18,52^ In brief, the interaction potential between the explicit molecules and their environment includes (a) a wall that confines the molecules within a rigid spherical cavity, (b) a reaction field (*U*_MF_) that describes the long-range interactions with bulk solvent, which are, in turn, partitioned into an electrostatic part and a nonelectrostatic part (*U*_MF_ = *U*_el_ + *U*_vW_). The former contribution is described by means of an implicit dielectric medium (here, the conductor-like polarizable continuum model, CPCM^53^), whereas the latter contribution is recovered by an optimization procedure. Since the purpose of *U*_MF_ is to avoid spurious boundary effects and deviations from bulk density in different layers of the spherical box, we used the bulk density as the target of the optimization of *U*_vW_. Additional details about the optimization procedure for nonaqueous solvents can be found in our previous report.^24^ The protocol starts with the division of the spherical cavity into Ng concentric shells and with the following definition of the *U*_vW_ term
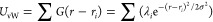
18where the index *i* runs on the concentric shells and *G*(*r*) represents a Gaussian function with constant spread (σ) and variable height (λ_*i*_). At predefined intervals, the average density in each concentric layer is compared to a threshold (the interval [−1.0025ρ,1.0025ρ] was used with ρ being the bulk density); the height (λ_*i*_) of each shell is increased or decreased by a fixed amount and *U*_vW_ is updated. The local densities will initially deviate from the target (bulk density) and slowly (after some tens of nanoseconds) converge to it. When a satisfactory convergence is reached, the corresponding profile is saved; since *U*_vW_ acts mainly near the border of the NPBC box, prior to the fitting, the profile is truncated once its value is below 0.1 kJ/mol. The potential energy profile is finally fitted to a polynomial expression
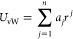
19The degree of the polynomial is determined running the corresponding ridge regressions. We tested degrees from 0 to 10, and for each degree of the polynomial, the shrinking factor value was optimized with a standard genetic algorithm (GA)^54^ with a population size of 50, a mutation rate of 0.3, and a crossover rate of 0.5 for 500 iterations. The degree of the polynomial was finally selected by choosing the best outcome of the corresponding learning curves for the root-mean-square error (RMSE) and *R*^2^ values.

### Treatment of the Embedding Effects in Quantum Mechanical Calculations

The foundation and implementation of multiscale QM/MM methods have been reviewed several times.^4,29,55−57^ Hence, only the general aspects relevant for the present contribution are briefly recalled.

In the ONIOM/EE approach, the complete system (referred to as real system) is partitioned into different fragments (referred to as model systems) described at different levels of theory. The, the electronic energy of the model system is computed explicitly accounting for the presence of the environment charge distribution by adding an electrostatic contribution in the Hamiltonian.

In the PMM approach, instead, environmental effects are considered as small perturbations tuning the Hamiltonian of the model system (usually referred to as quantum center, QC, in this context) built on the eigenstates of the unperturbed model system. The perturbing contributions correspond to the electric field originated from the atomic charges of the solvent atoms in the different configurations obtained from an MD simulation. In the original implementation of the method (QC-based expansion), the perturbing electric field is expanded around a single QC reference position (typically the center of mass). Then, a more refined model was proposed (atom-based expansion, employed in the present work),^38^ in which the perturbation is expressed in terms of the electric field generated by the solvent at each QC atom. Diagonalization of the resulting perturbed Hamiltonian matrix provides the instantaneous perturbed electronic eigenstates for a given QC-environment semiclassical configuration. Interested readers can refer to the literature^34,35,37,38^ for further details.

In the integrated ONIOM/EE-PMM approach,^39,41^ the complete classical sampling is at first analyzed to identify a set of relevant basins or clusters for partitioning the trajectory. Then, for each of these subsamplings, a single reference configuration is selected for performing ONIOM/EE computations. Next, the PMM is applied within each basin to treat the fluctuations of the perturbing environment by expressing the perturbed Hamiltonian matrix on the basis of the ONIOM/EE eigenstates computed for the reference configuration. At the end, for each snapshot of each subsampling, the procedure provides perturbed eigenstates and energies that, collected together, allow the reconstruction of the relevant features of the system resulting from the complete trajectory.

### Computational Details

#### MD Simulations

NPBC simulations were run with a locally modified version of the Gaussian^58^ suite of programs. The RVV1 integrator was used in all simulations, with an ϵ = 10^–9^ convergence criterion for the calculation of quaternion derivatives. The van der Waals mean-field potential for acetonitrile was optimized employing a system composed of 382 solvent molecules enclosed in a spherical nanodroplet with a radius of 20 Å (with the bulk solvent, treated by the conductor version of the polarizable continuum model, CPCM^53^ starting at 22 Å). This system was simulated in an NVT ensemble for 5 ns at 300 K (Berendsen coupling scheme) with a time step of 2.0 fs, starting from random positions of the molecules. The coordinates of the last configuration of this trajectory were used for short NVE simulations (1 ns) with varying time steps (0.5, 1.0, 2.0, and 4.0 fs, respectively) to assess the stability of the RVV1 integrator. Then (under the same conditions, with δ*t* = 2.0 fs), the GLOB optimization procedure was carried out for 20 ns, updating the Gaussian profile every 50 000 steps. The “rough walls” boundary condition was used in all NVT/NPBC simulations. The cavity was divided into bins of 0.25 Å (Ng = 81 density layers) to optimize and use the MF term. Once a stable MF potential was obtained, solute/solvent simulations were carried out embedding each solute in a spherical solvent cavity with a radius of 20 Å and centered at the solute center of mass. The equilibration of the system involved an initial minimization with the conjugate gradient method and a subsequent simulation for 1000 ps with a small integration step of 0.5 fs and temperature of 298.15 K. The production run was then initiated at 298.15 K and continued for 25 ns with an integration time step of 2.0 fs. Snapshots were saved at 2 ps interval, and the last 20 ns were used for post-processing. Tyrosine bond lengths were kept fixed by means of the RATTLE^59^ method, which was also implemented in our MD engine.

#### Clustering

**Figure 1:**
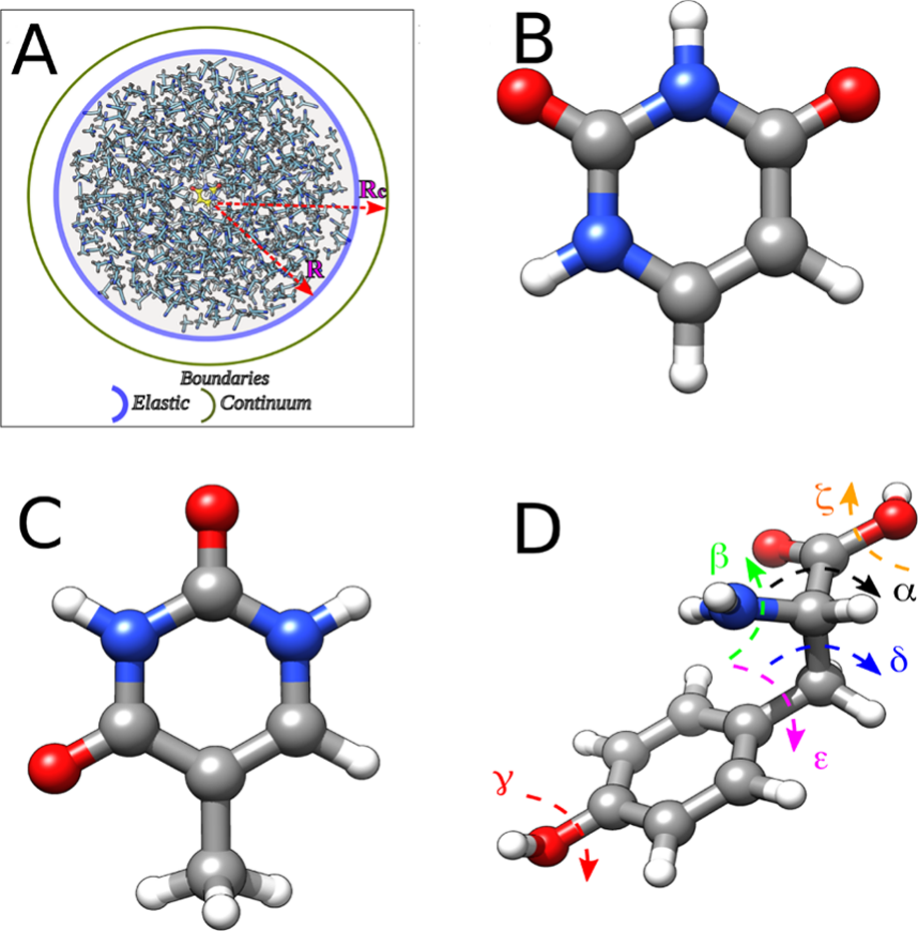
(A) Schematic drawing of the GLOB model. (B–D) Ball-and-stick representation of the chromophores studied in this contribution: (B) uracyl, (C) thymine, and (D) tyrosine; the dashed arrows and Greek letters represent the degrees of freedom used for dihedral principal component analysis (DPCA) and clustering (see Figures 12 and 14).

In this study, we need: (i) a good representative point (centroid) for each cluster, which will be used as the reference configuration for the solute in the following ONIOM/EE calculations and (ii) a robust and general recipe to assign similar structures to the same cluster to apply the PMM procedure. Since inclusion or exclusion of a single MD frame has a negligible effect on the computational cost for treating in-cluster fluctuations, we did not see particular advantages in using density-based methods (e.g. DBSCAN^60^), which give a division between “real ” and “noise” points. For analogous reasons, the precise assignment of simulation frames with intermediate structures is scarcely relevant since each classical frame gives a tiny contribution to the overall signal. For all of these reasons, simulation frames were clusterized by the simple yet effective partition around medoids (PAM) algorithm,^61^ which also allows a straightforward implementation of internal validation methods to PAM runs.^62^ To determine the best number of clusters (*k*), we run PAM for values from 2 to 20 and then used the Silhouette score (SI) and Dunn index^62^ (DI) internal validation criteria to determine the best *k* in addition to looking for a breakeven point in the within sum of squares error (WSS). Both SI and DI should have a maximum corresponding to the parameter set (just the value of *k* in this case) that yields the best clustering, while for WSS, one looks for a change in the slope. Hence, the best value of *k* was obtained from the consensus of three independent criteria. Dihedrals would be a sensible choice for the feature space but cannot be used directly because of torsional periodicity. Therefore, we used the dihedral principal component analysis (DPCA) approach (thus switching from a 6- to a 12-dimensional feature space; see Figure 1B) described by Altis et al.,^63^ which also allowed us to reduce the number of features to be used in dissimilarity calculations; we chose the minimum number of principal components, which yield 90% or more of the original variance. After having obtained a reduced feature space, we used the so-called L∞ or Chebichev distance^62^ to compare structure pairs, to maximize the dissimilarity between structures having a different orientation in one of the transformed coordinates.

#### ONIOM/EE-PMM Calculations

The whole solute was always taken as the QC (i.e., the model system), whereas the solvent molecules represented the perturbing embedding environment. The dependence of the electronic properties of a semirigid QC from its structural deformations can be generally treated, if needed, a posteriori by means of harmonic QM models introducing vibronic contributions by means of Franck–Condon and Herzberg–Teller models.^41^ As a consequence, the classical sampling can be safely performed by keeping the solute constrained in its equilibrium structure. Furthermore, in the present context, only low-resolution experimental spectra are available so that we can disregard additional vibronic computations. We have shown^39,41^ that in the case of rigid solutes not experiencing too strong solute–solvent interactions, the complete trajectory can be considered as a single extended basin. Therefore, we performed just one expensive ONIOM/EE computation, then applying the PMM for all of the remaining frames of the trajectory.^39,41^ At variance, to deal with flexible QC, the approach followed in previous studies^39^ was further improved. In fact, the former procedure was based on a sort of “visual partitioning” in which the trajectory is divided into four subtrajectories according to the value of the dihedral angle defining the orientation of the hydroxyl group with respect to the aromatic ring. In the present context, we performed, instead, an unsupervised cluster analysis based on internal coordinates to identify both the set of clusters composing the trajectory and the cluster centroids. However, the issue of the selection of the reference structures (used for ONIOM/EE computations) was not completely solved by taking the most representative solute conformation from each cluster. Thus, for each cluster, we followed a procedure reminiscent of the so-called ASEC method,^64,65^ employing a “collective frame” representative of the average configuration of the molecular environment for the corresponding cluster by extracting 30 snapshots sequentially from each subtrajectory and assigning 1/30 of the actual atomic charge to each environmental atom for the ONIOM/EE calculations.^41^ We computed the first 11 electronic states and the complete matrix of the corresponding dipole moments by exploiting the ONIOM/EE model by means of time-dependent density functional theory (TD-DFT) using the B3LYP^66^ functional with the 6-311G(d) basis set. For each electronic state, the corresponding atomic charges were also computed according to the CM5 methodology by employing the same level of theory.^67^ All of the relevant QM data were utilized to apply the perturbative approach for evaluating environmental effects beyond the reference configurations. Gaussian distribution functions were used as broadening functions to get the absorption spectra for all of the basins. We used the same sigma value employed in previous studies on tyrosine (0.0008 au of frequency).^36,39^ The final absorption spectrum is then obtained by weighting the spectra resulting from each basin according to the corresponding cluster population. For the case of uracil and thymine, we exploited the solute structural rigidity and the lack of strong solvent effects to apply the procedure according to a very simple yet effective scheme.^39^ Namely, we computed from the corresponding MD simulations the values of the three components of the electric field acting on the center of mass of the solute due to the solvent molecules. Then, from each trajectory, the MD frame characterized by the electric field components closest to the average values was extracted to be utilized as the reference configuration. On each selected configuration, the first 11 unperturbed electronic states and the complete matrix of the corresponding dipole moments were computed using the TD-DFT theory (CAM-B3LYP/6-311+G(d)) within the ONIOM/EE procedure. Then, fine tuning of the spectra by fluctuations within each cluster was taken into account through the perturbative approach.

## Results and Discussion

### Analysis of MD Trajectories

#### Stability of the Rigid-Body Integrator

In this section, we analyze the stability of the RVV1 integrator when simulating pure CH_3_CN nanodroplets under NPBC by running several simulations with increasing time steps. The general robustness of the quaternion-based approach for propagating the equations of motion has been extensively tested in previous studies for TIP4P water^48−50^ systems under PBC; hence, our purpose here is to fully assess the integrator stability under different conditions (e.g. for a system with a higher number of long-range interactions compared to TIP4P water). To this end, prior to the optimization of the MF potential, we started a set of 1 ns long NVE simulations of pure CH_3_CN with time steps of 0.5, 1.0, 2.0, and 4.0 fs.

**Figure 2:**
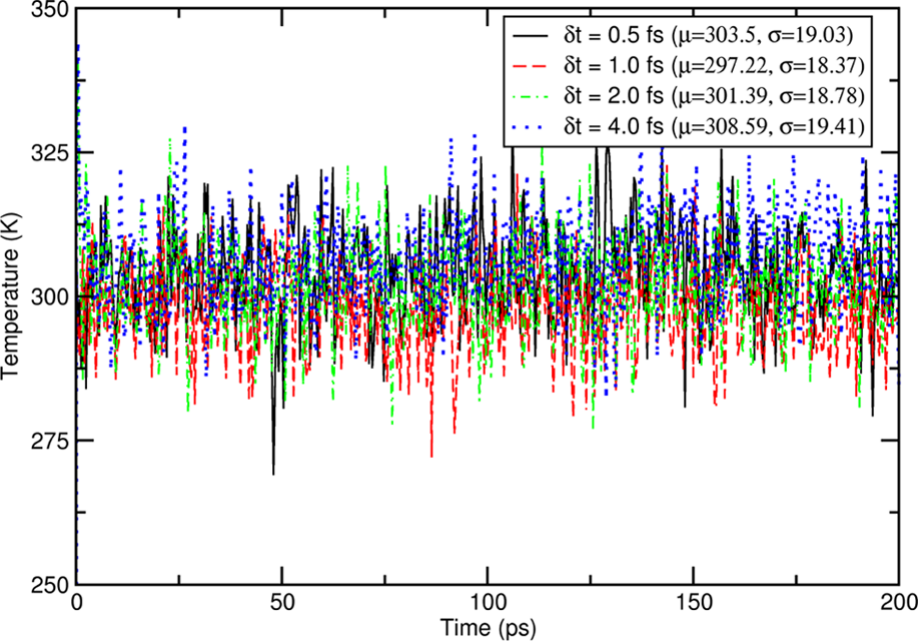
Time evolution of the temperature in the last 200 ps (after equilibration) of an NVE simulation under NPBC of pure CH_3_CN; the labels show the different time steps used and the mean and standard deviation for each trajectory.

**Figure 3:**
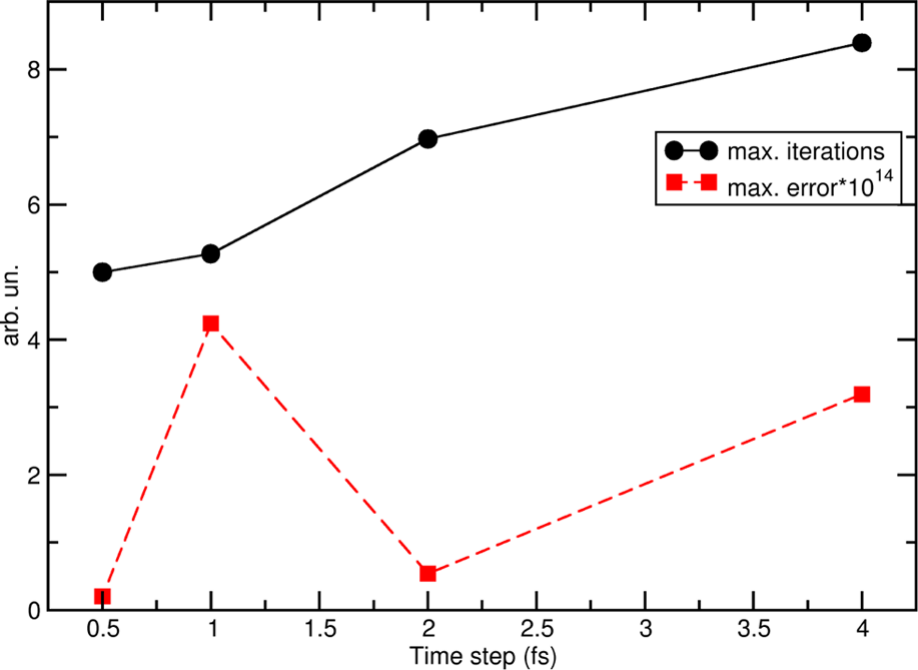
Average maximum number and convergence error of pure CH_3_CN trajectories under NPBC as a function of the integration time step. The averages were calculated for the last 200 ps of each simulation.

Figure 2 shows the temperature of the last 200 ps of the NVE trajectories for the various time steps; it is quite apparent that systematic drifts are never present, in line with previous results. Figure 3 shows the average number of self-consistent iterations in the first part of the RVV1 algorithm needed to achieve the desired accuracy of 10^–13^ and the corresponding error obtained on the last 200 ps of each trajectory; note that this average is calculated on the maximum value of iterations performed for any given rigid body in each time step and, coherently, the reported error is an average of the highest values obtained in each simulation step. The results are quite similar to those obtained by Rozmanov et al. for TIP4P water: less than six iterations (on average) are needed for time steps of 0.5 or 1.0 fs, and about eight iterations for larger time steps, the higher error obtained for δ*t* = 1.0 fs with respect to 2.0 and 4.0 fs being related to the reduced number of iterations performed.

**Figure 4:**
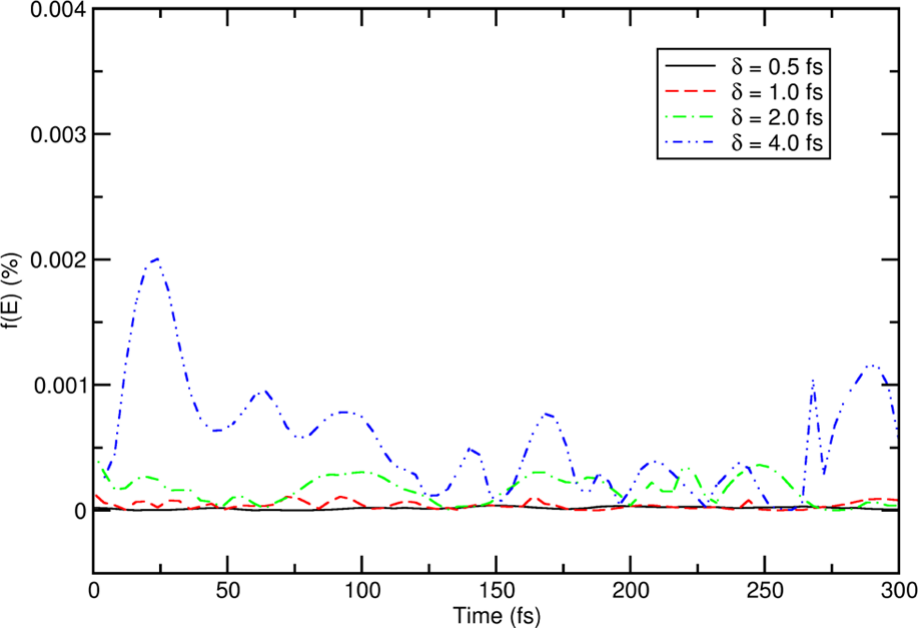
Total energy fluctuation (percentage) for the first 300 fs of pure acetonitrile simulations.

Finally, Figure 4 shows the total energy fluctuation for the first 300 fs of various runs, which was calculated as a percentage of , where ⟨*E*⟩_*t*_ is the average energy. It is quite apparent that the run corresponding to δ*t* = 4.0 fs shows larger oscillations of the total energy compared to simulations with smaller time steps (but still in the range of 10^–6^ with respect to the total energy), a behavior also observed for TIP4P water. Given the high accuracy that we seek for computational spectroscopy applications and the relatively small computational cost of these MD simulations, we choose a time step of 2.0 fs for all NPBC simulations.

#### Optimization of MF Potential for Acetonitrile

**Figure 5:**
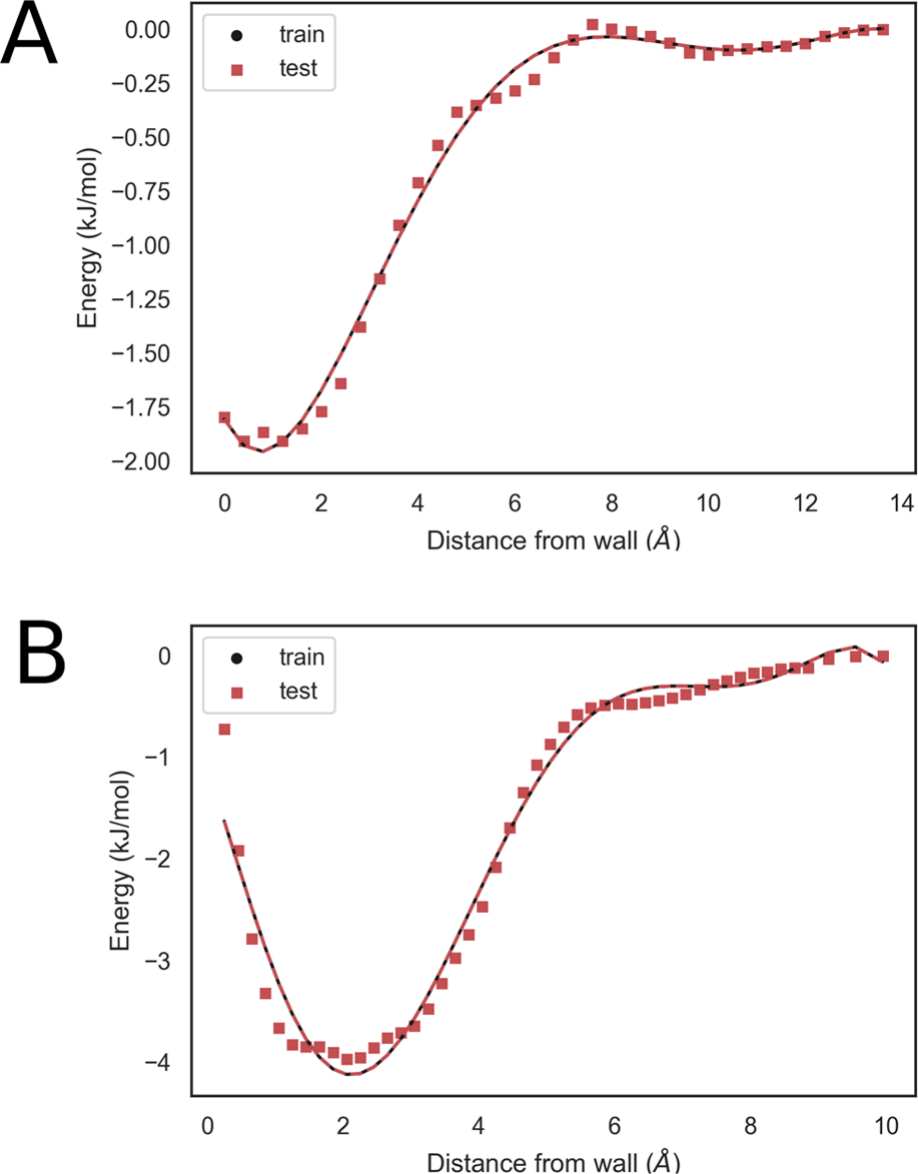
Mean field potential energy profile; data used in the fitting and the corresponding fitted polynomial are drawn in black, while the test points are drawn in red. (A) Results obtained for methanol and (B) results obtained for acetonitrile.

**Figure 6:**
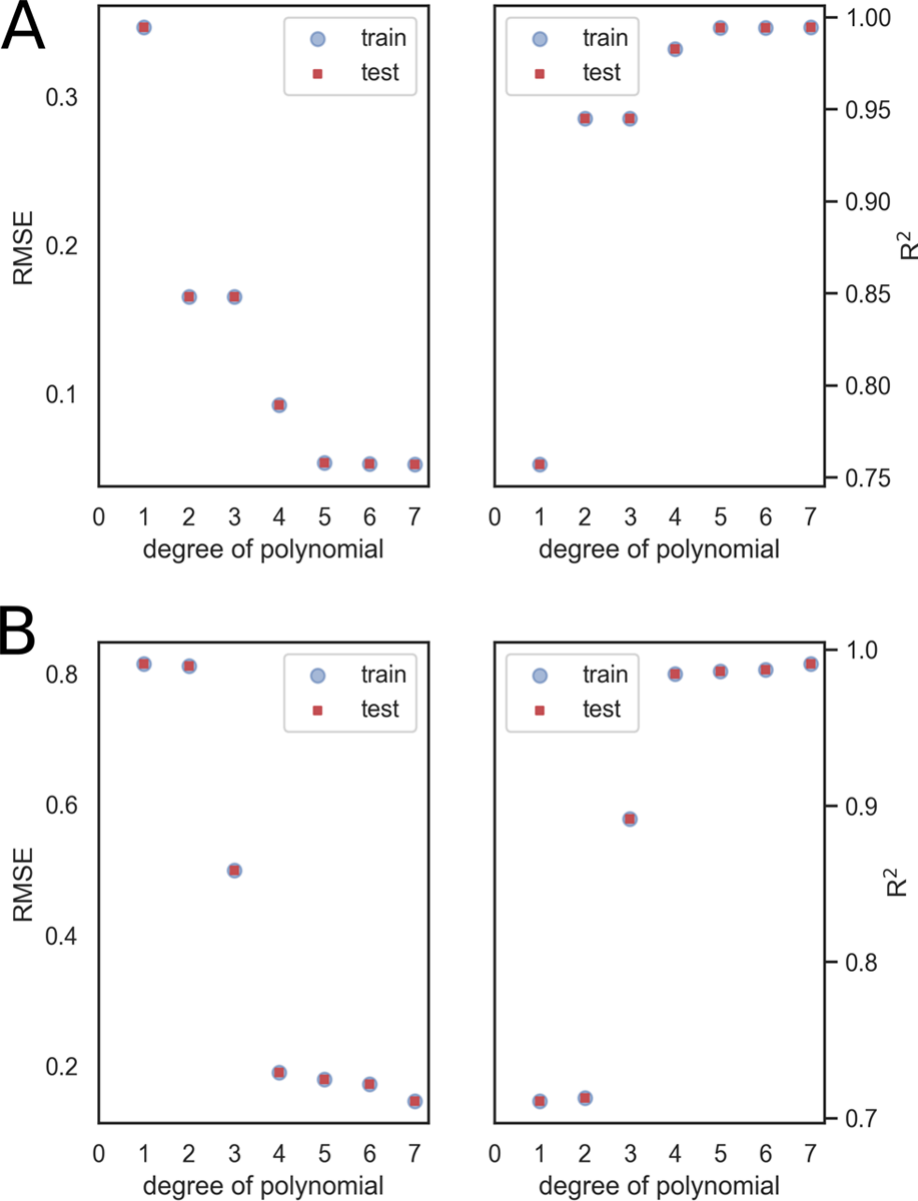
Value of RMSE and *R*^2^ as a function of the degree of the polynomial; train and test values are superimposed. (A) Results obtained for methanol; (B) results obtained for acetonitrile.

Once the stability of the rigid-body integrator for the acetonitrile nanodroplets was assessed, we run a 40 ns long simulation to obtain an optimized *U*_vW_ mean-field potential energy profile using the simulation settings described in the section devoted to computational details. The obtained profiles are shown in Figure 5 (without the truncated portion) together with the fitted polynomial. The fit was carried out over 50 points with a resolution of 0.2 Å, and the test set was obtained in the same way. Looking at the learning curves for RMSE and *R*^2^, we selected a fifth (for methanol)- or fourth (for acetonitrile)-degree polynomial to fit *U*_vW_, and the corresponding parameters are shown in Table 1 and in Figure 6.

**Table 1:** Parameters of the *U*_vW_ Polynomial Fits for Methanol and Acetonitrile

parameter	CH_3_OH	CH_3_CN
*a*_0_	–1.8081	7.4319 × 10^–3^
*a*_1_	–4.3732 × 10^–1^	–3.8878
*a*_2_	3.5936 × 10^–1^	1.4842
*a*_3_	–6.2067 × 10^–1^	–1.8302 × 10^–1^
*a*_4_	4.2625 × 10^–3^	7.1257 × 10^–1^
*a*_5_	–1.0404 × 10^–4^	NA

**Figure 7:**
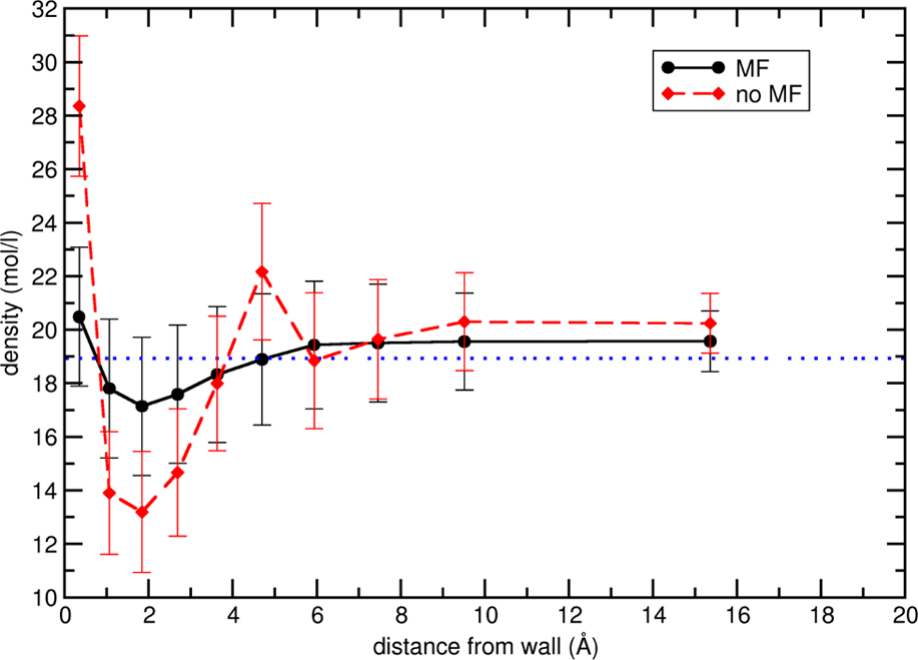
Average density and the corresponding standard deviations of CH_3_CN in 10 concentric shells of constant volume for NPBC CH_3_CN simulations. The results in the presence (black circle full line) and absence (red squares and dashed line) of *U*_vW_ mean field are compared to the total box density (blue dotted line).

Finally, the effect of the presence of the optimized *U*_vW_ term on the acetonitrile box was assessed running a final 5 ns simulation and plotting the average density in concentric spherical shells of constant volume, as shown in Figure 7. Inclusion of the *U*_vW_ contribution leads to a maximum deviation from the bulk density of about 1 mol/L at 2 Å from the wall and to a stable density at 6.0 Å from the wall, whereas the profile without *U*_vW_ shows larger deviations and reaches a stable value at a longer distance from the wall.

Methanol was already parameterized for use within GLOB in a previous paper.^24^ Here, we have refitted the original data, obtaining well-converged results for a fifth-degree polynomial, whose coefficients are given in Table 1.

### Application of the ONIOM/EE-PMM Procedure

#### Rigid Solutes: Uracil and Thymine

**Figure 8:**
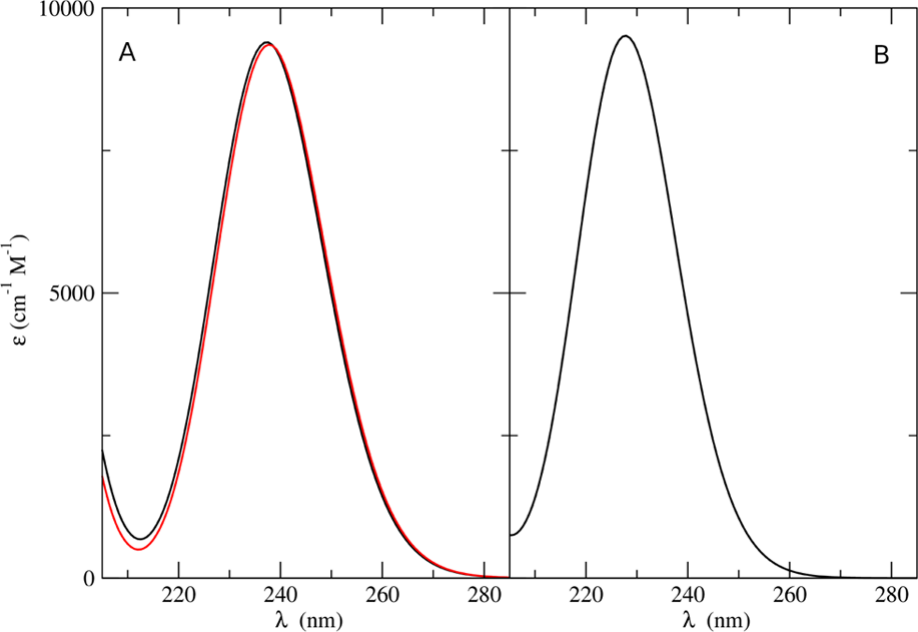
(A) UV absorption spectra of thymine in acetonitrile obtained from ONIOM/EE-PMM (black line) and ONIOM/EE (red line) procedures. (B) ONIOM/EE-PMM spectrum of uracil in acetonitrile.

We computed the UV–vis absorption spectra of thymine and uracil in acetonitrile according to two of the QM/MM procedures outlined above, namely, the conventional ONIOM/EE method and the integrated ONIOM/EE-PMM approach. Within the approximation of rigid solute MD sampling, we employed around two hundred equispaced snapshots to perform QM calculations when employing the ONIOM/EE procedure. Test computations confirmed that this number of snapshots is largely sufficient to obtain well-converged spectra and, indeed, lower numbers of snapshots (around 100) are normally sufficient. On the other hand, for the ONIOM/EE-PMM procedure, we utilized only one structure for the QM calculations. The results shown in Figure 8A demonstrate that application of both the proposed methods produced almost identical results, in good agreement with the experimental data. In fact (besides the typical shift characterizing the level of theory of the electronic calculations), from all of the computations, we obtained spectra characterized by one peak in the 200–300 nm region with a maximum absorption coefficient of about 9000 M^–1^ cm^–1^ and a full width at half-height (FWHW) of ≈0.6 eV (the experimental values are: λ_max_ = 261 nm, FWHM = 0.6 eV).^68^Figure 8B shows the UV absorption spectra of uracil in acetonitrile obtained by the ONIOM/EE-PMM procedure. The spectrum shows again a peak in the 200–300 nm region with a maximum absorption coefficient of about 9000 M^–1^ cm^–1^ and a full width at half-height of ≈0.6 eV, in line with the experimental results.^68^

**Figure 9:**
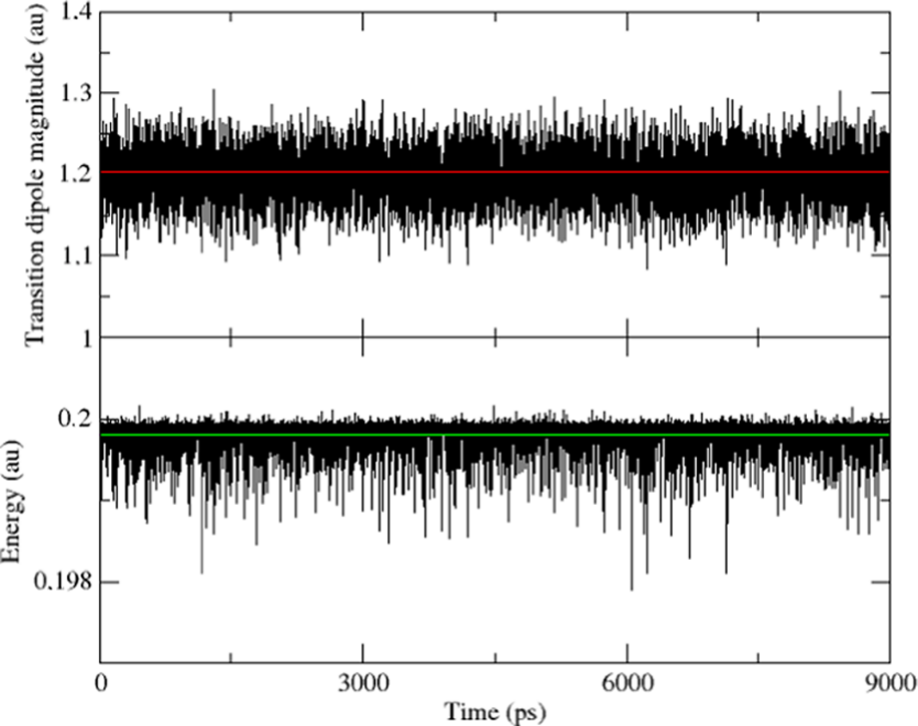
Comparison between the results of the single ONIOM/EE calculation (colored line) and the complete PMM outcome (black line) for the transition dipole moment (top) and the transition energy (bottom) obtained for uracil in acetonitrile.

To explicitly address the effect of the solvent fluctuations modeled by the PMM, in Figure 9, we report a comparison between the single ONIOM/EE calculation (the reference) and the complete PMM outcome for the transition energy and the transition dipole moment of uracil in acetonitrile. It is quite apparent that both quantities fluctuate around their ONIOM/EE values with oscillations small enough to be confidently described by a perturbative approach. From the perspective of the general procedure, inclusion of the PMM treatment of the fluctuations within each cluster (following the trajectory partitioning) allows us to avoid the customary practice of simulating these effects by a phenomenological Gaussian broadening with a negligible computational cost.

**Figure 10:**
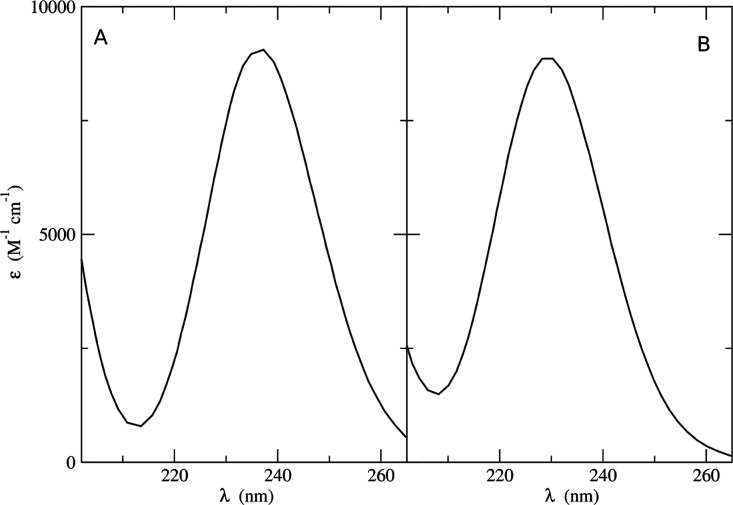
UV absorption spectra of (A) thymine and (B) uracil in methanol obtained from ONIOM/EE-PMM computations.

We then computed the UV–vis absorption spectrum of thymine and uracil in methanol according to the ONIOM/EE-PMM procedure, employing again just one structure for the full QM/MM calculations. The spectra shown in Figure 10 are again in satisfactory agreement with the experimental results.^68^

**Figure 11:**
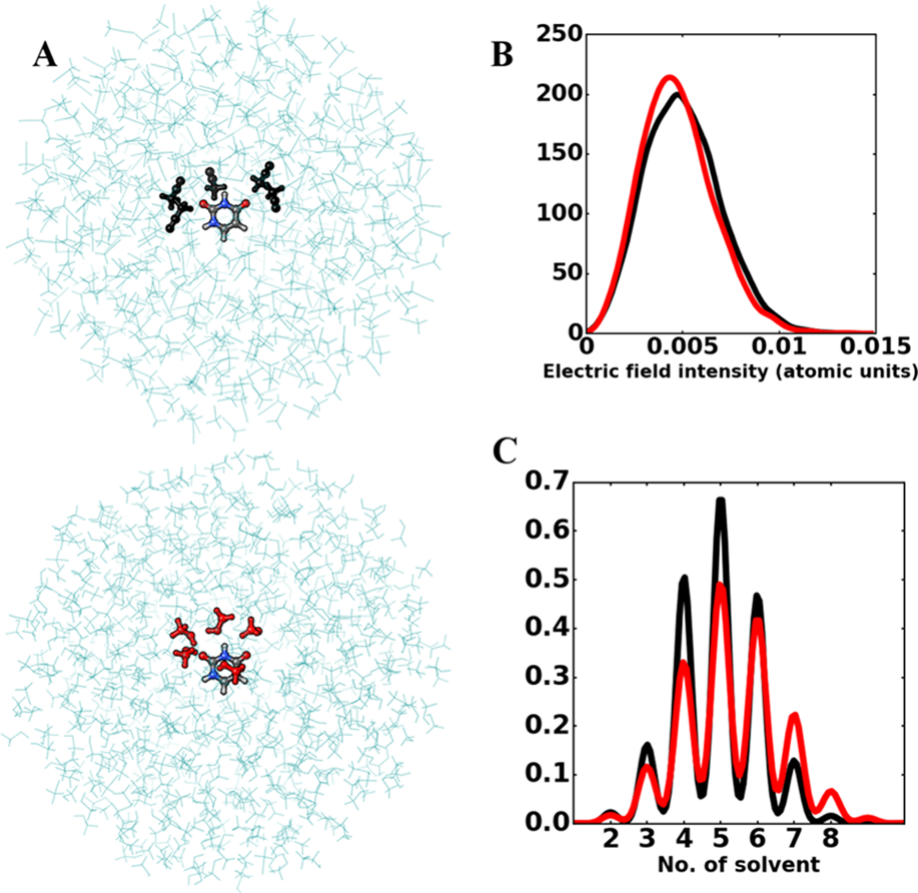
(A) Representative snapshots of uracil in acetonitrile (top) and methanol (bottom) solutions. Solvent molecules within 3 Å from uracil oxygen atoms are shown in a ball-and-stick mode. (B) Distribution of electric field intensities exerted by acetonitrile (black line) and methanol (red line) on uracil. (C) Distribution of the number of solvent molecules within 3 Å around uracil.

A comparison of the spectra obtained in methanol and acetonitrile shows that the solvatochromic shifts originated from these two solvents are comparable, in agreement with the tiny displacement of the absorption maximum (about 0.05 eV) shown by the corresponding experimental spectra.^69^ As a matter of fact, from our MD simulations, we inferred that embedding uracil in methanol or acetonitrile does not entail dramatic differences when the electric field exerted on the solute by the environment is considered. In support of this, in Figure 11B, the distributions of the electric field intensity exerted on uracil center of mass in both solutions are shown. Likewise, inspection of the uracil solvation shell showed that, on average, four to five solvent molecules can be found close to the uracil oxygen atoms (precisely, within a sphere of 3.1 Å centered on each oxygen) irrespective of the simulated solvent. The distributions are shown in Figure 11C along with representative snapshots of uracil in both solutions, where the solvent molecules close to the solute are highlighted.

#### Flexible Solute, Step 1: Clustering and Determination of the Reference Structures

**Figure 12:**
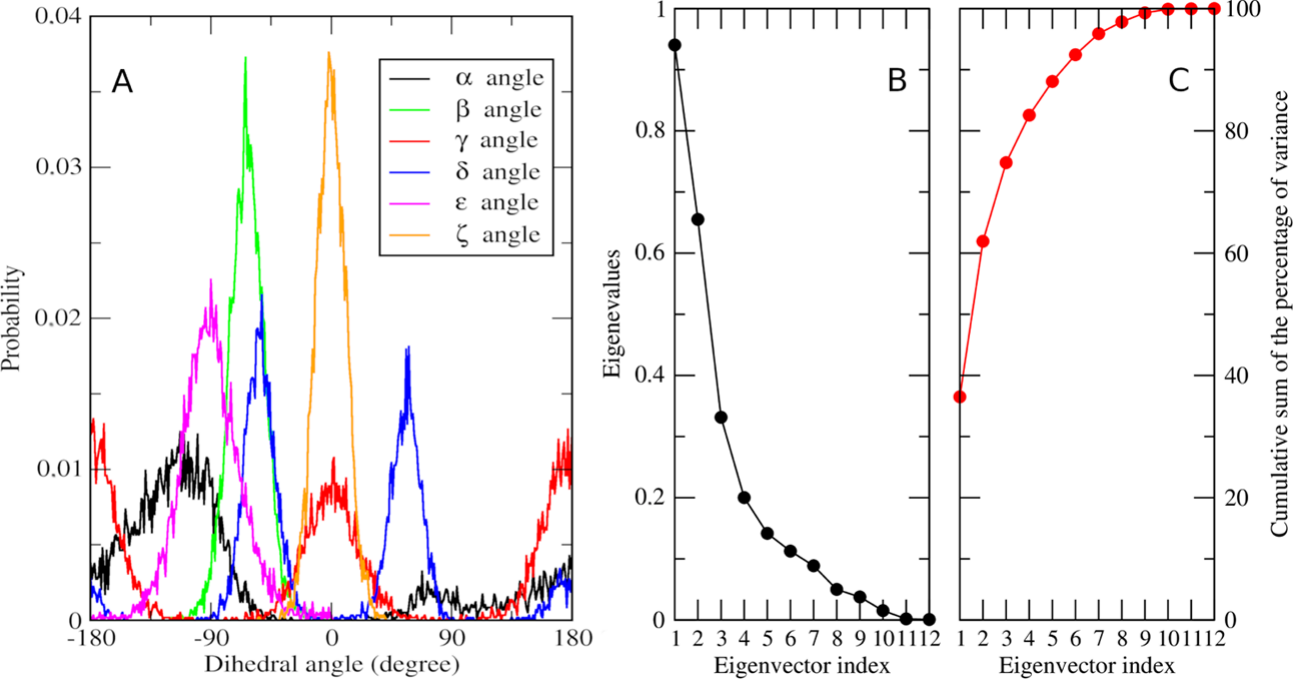
(A) Distribution of the dihedral angles in tyrosine sampled by the MD simulation in acetonitrile (refer to Figure 1 for labels and colors). (B, C) Results of the DPCA: amount of variance explained by each of the selected components (namely, the eigenvalues of the covariance matrix, black dots) and cumulative sum of the percentage of variance explained by each of the selected components (red dots).

A rational way of analyzing the conformational space explored by a flexible solute of average size during a simulation is by computing the distribution of the sampling for each dihedral angle. In fact, the profiles provide a “visual” idea of both the conformations possibly assumed by the molecules and the least probable ones, as shown in Figure 12, for the case of neutral tyrosine in acetonitrile. For most of the angles, the distributions follow the expected sinusoidal trend, except for the α angle where the higher probability of having the carboxyl hydrogen close to the amino group is reflected by the asymmetry of the curve. Note that for β and ζ angles (see Figure 1 for labeling), which represent the rotations of the amino group and the benzene ring, respectively, we initially obtained multimodal profiles. These collapsed into the curves reported in the figure after removing redundancies due to atom types symmetry. Correlation among the dihedral angles was analyzed by means of DPCA. Given the periodicity of the six dihedral angles, the corresponding sine and cosine values were computed, thus obtaining the12-dimensional space used for computing the covariance matrix. The results presented in Figure 12B,C show that structural deformation along the first four principal components cover around 80% of the tyrosine internal motion and that the 90% threshold is reached with six components. To exploit the advantage of using internal coordinates, we utilized the space spanned by the first 10 principal components (this corresponds to 99% of the original variance) to define the feature space to be clustered (see the Methods section for details).

**Figure 13:**
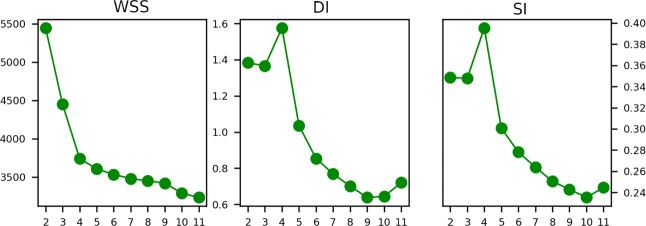
Within-cluster sum of squares error (WSS), Silhouette coefficient (SI), and Dunn index (DI) computed for different number of clusters.

To get the best number of clusters, we run the PAM procedure from *k* = 2 to *k* = 20 and calculated the corresponding validation criteria: WSS, DI, and SI. The results shown in Figure 13 show without ambiguities that *k* = 4 is a good value to partition data.

**Figure 14:**
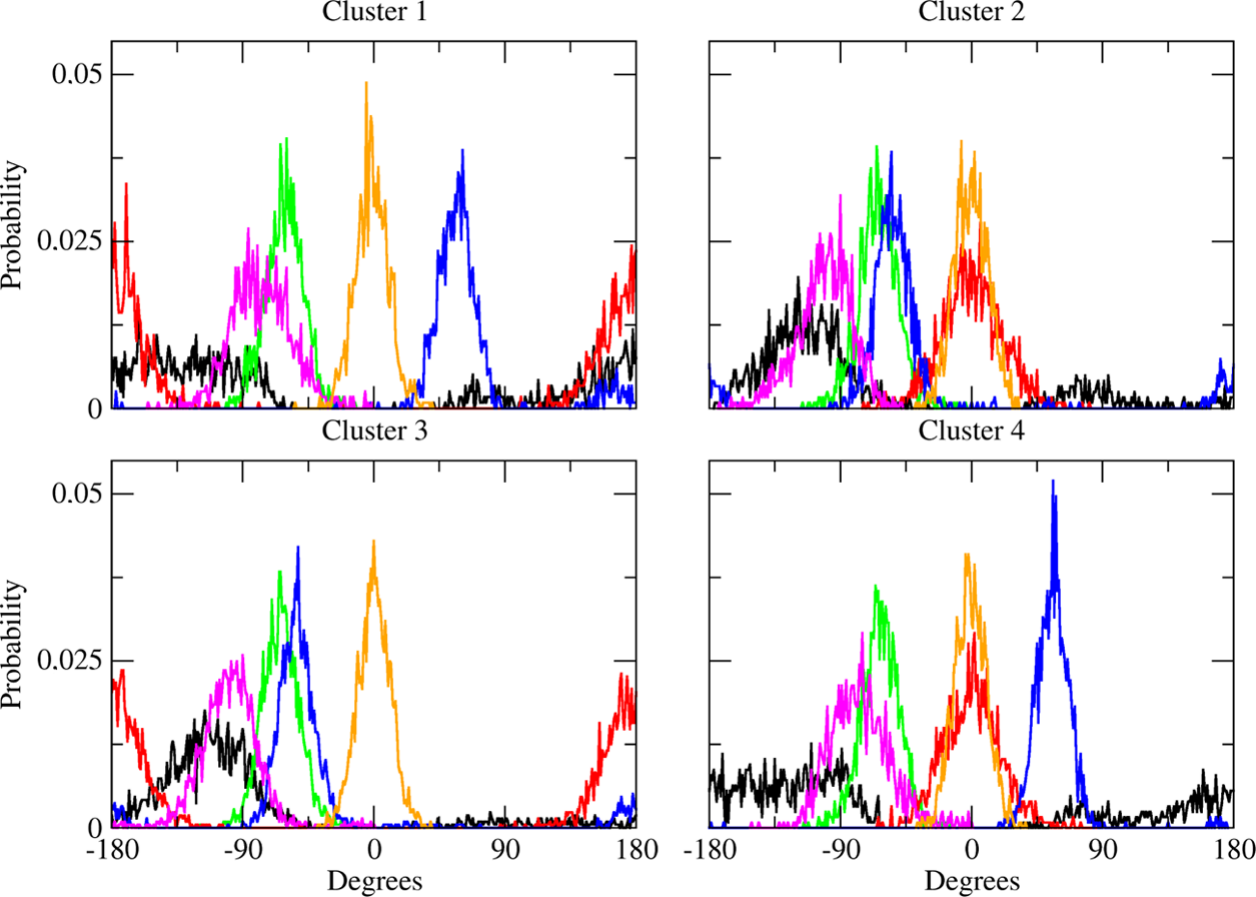
Distributions of the dihedral angles in tyrosine sampled within each cluster (α: black line, β: red line, γ: green line, δ: blue line, ε: yellow line, ζ: cyan line; refer to Figure 1 for the labeling of the dihedral angles).

With this in mind, we divided our trajectory into four clusters. In an attempt to get insights into the nature of the partitioning, we computed the distributions of tyrosine dihedral angles within each cluster, as reported in Figure 14. Comparing this with Figure 12A allows us to catch the nature of the partitioning. In fact, almost all of the profiles (again except that of α) turned into unimodal distributions. As outlined in the Methods section, the clustering procedure provides also the centroid of each cluster. In Table 2, we report the conformational features of these structures along with the percent weight of each cluster in the total sampling. Shifting the comparisons to Cartesian coordinates, in Table 3, we report the RMSD obtained by pairwise comparing the centroids and the in-cluster average RMSD (computed with respect to the corresponding centroid). These data also show that when taking into account a slightly different feature (clearly related to the one used for the clustering), a satisfactory partitioning of the starting trajectory is obtained.

**Table 2:** Values of Dihedral Angles of Each Cluster Centroid (Refer to Figure 1 for Labeling) and Percent Weight of Each Cluster in the Total Sampling

	centroids dihedral angles (degree)						
	α	β	γ	δ	ε	ζ	statistical weight (%)
cluster 1	–163	–71	–177	71	–80	2	20
cluster 2	–119	–65	0	–57	–96	6	21
cluster 3	–116	–61	177	–63	–103	4	37
cluster 4	–156	–66	9	68	–75	–3	22

**Table 3:** Results of RMSD Analyses: Average In-Cluster RMSD with Respect to the Corresponding Centroid in the Diagonal Elements of the Table (Also Highlighted in Bold), Centroids Pairwise RMSD in the Remainders^a^

	cluster 1	cluster 2	cluster 3	cluster 4
cluster 1	**0.57**	1.58	1.57	0.22
cluster 2		**0.57**	0.23	1.63
cluster 3			**0.50**	1.63
cluster 4				**0.51**

a The values are reported in Å.

#### Flexible Solute, Step 2: Spectroscopic Calculations

**Figure 15:**
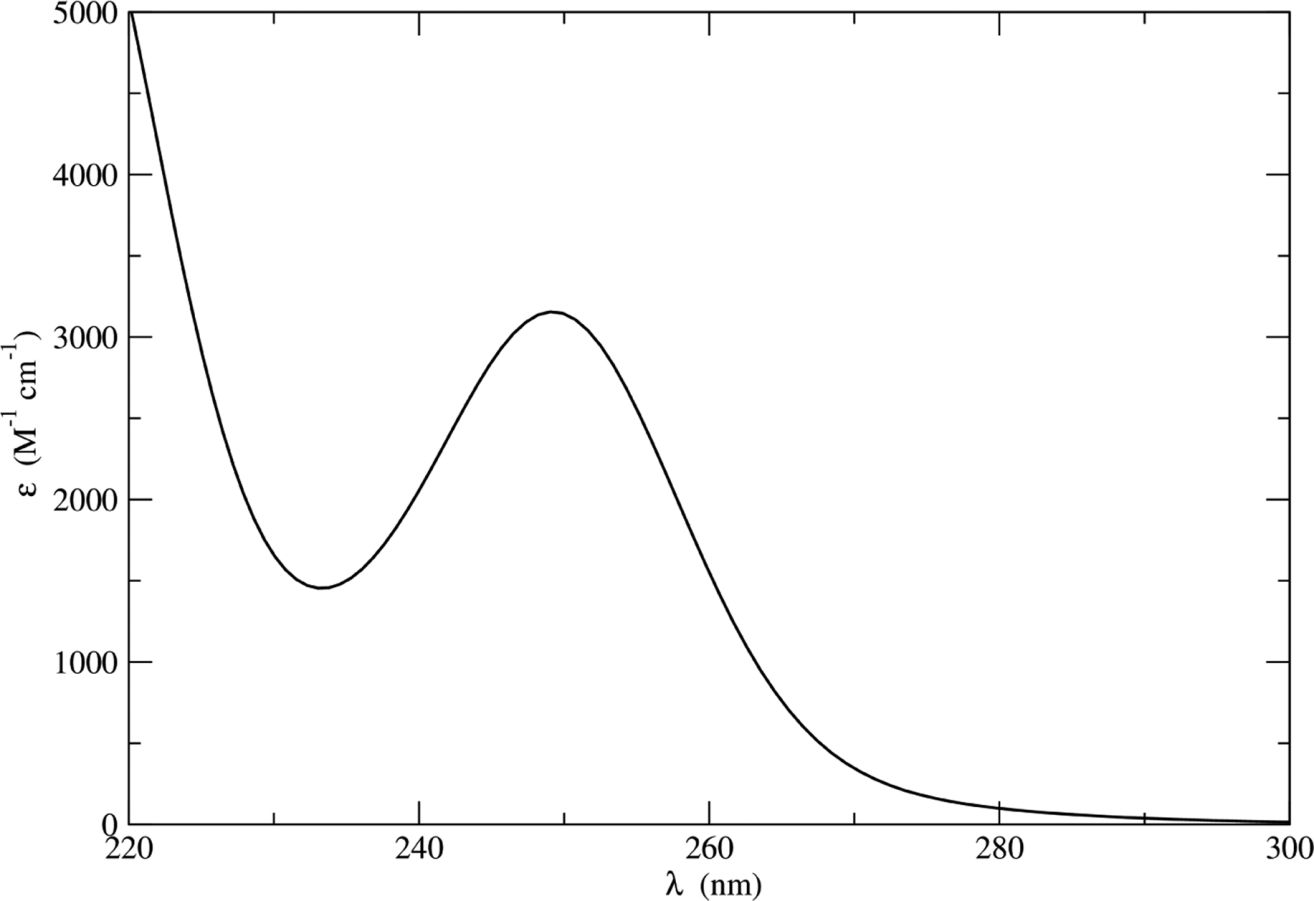
Tyrosine in acetonitrile electronic absorption spectrum as obtained by applying the ONIOM/EE-PMM procedure.

The centroids of the four clusters defined above were extracted from each subtrajectory and then utilized for QM calculations. Since the whole clustering procedure concerned only the tyrosine internal (classical) motion, the selected structures needed to be properly complemented with representative arrangements of the embedding solvent to be used for the reference ONIOM/EE calculations (see the Methods section). To this end, for each cluster, we composed a collective frame by putting together different instantaneous solvent configurations, while for the following EE calculations, we scaled the charge of each solvent atom according to the number of configurations collected. This way, we provided the references representation of each average in-cluster solute–solvent interaction. Then, the corresponding local fluctuations were modeled with the PMM. Namely, we applied the procedure by treating each cluster as the simulation of a semirigid solute and then by weighting the outcome of each single-cluster calculations by the statistical relevance of the corresponding cluster, as reported in Table 2. Application of the procedure provided the spectrum reported in Figure 15. Experimentally, the absorption spectrum of the tyrosine zwitterion has been recorded in aqueous buffer solution presenting an absorption peak around 277 nm characterized by an extinction coefficient of around 1400 M^–1^ cm^–1^.^70,71^ Conversely, to model tyrosine within proteins, the related peptide analogue Ac-Tyr-NH_2_ can be studied. The absorption spectrum in acetonitrile of this chromophore, more closely resembling our solute, is characterized by a peak around 278 nm of 1150 M^–1^ cm^–1^ intensity.^72^ Given the inherent difference between the simulated chromophore and the one experimentally studied, a quantitative agreement between the QM calculations and experiment cannot be expected, but, qualitatively, the computed spectrum appears fully reasonable.

A last comment is in order about the performances of the integrated ONIOM/EE-PMM approach in comparison to those of the standard ONIOM/EE and PMM models. According to previous results,^39^ for rigid solutes, the three models provided similar results. However, for flexible solutes, the standard PMM model provided disappointing results, whereas the integrated procedure was in very good agreement with ONIOM/EE at a strongly reduced computational cost (by about 2 orders of magnitude). In fact, the integrated procedure merges the strengths of the variational and perturbative methods. As a matter of fact, the variational procedure ensures the accuracy of the evaluation of the embedding effects on the electronic properties of the quantum portion of the system. Then, the perturbative approach provides a reliable description of the further fine tuning of the spectra by the fluctuations of the embedding environment overcoming the need for a huge number of calculations. Thus, the difficulties faced by conventional methods (high cost of variational approaches and limited convergence radius of perturbative approaches) are avoided and the computational cost/accuracy ratio is cut down. An accurate spectrum of tyrosine in acetonitrile can be obtained by means of just four full QM/MM computations in place of the 400–800 calculations required by the conventional ONIOM-EE approach.

## Conclusions

In the present contribution, we outlined the general workflow under active development in our laboratory for the spectroscopic characterization of chromophores in condensed phases. We focused our attention on two aspects: (i) the performance of a new RB MD integrator (based on quaternion representation) into the latest development of the MD engine within a modified version of the Gaussian software and (ii) the effectiveness of the ONIOM/EE-PMM strategy in conjunction with a clustering procedure to address both rigid and flexible chromophores within a general model enforcing nonperiodic boundary conditions. In fact, the stability of GLOB MD simulations confirmed both the validity and robustness of the molecular mechanics machinery employed. The obtained classical samplings were then utilized as the statistical ensembles to perform computational spectroscopy studies merging variational and perturbative approaches.

Seen as a whole, the proposed computational procedure (starting from NPBC simulations and then proceeding with clustering and ONIOM/EE-PMM computations) significantly enhances the feasibility of spectroscopic applications in condensed phases. From the one side, only the essential degrees of freedom are explicitly sampled and, from the other side, the number of expensive high-level computations is strongly reduced without any significant accuracy loss, but with the possible gain of additional insights from a simplified view.

In conclusion, we think that, with further developments and validations underway, we have already developed an effective tool for aiding the assignment and interpretation of electronic spectra of medium-size chromophores in condensed phases. Extension to realistic models of biological systems requires the effective treatment of chromophores embedded in a macromolecular chiral cavity rather than in a substantially isotropic solvent.^73^ While both ONIOM and PMM can, in principle, deal also with these situations, proper tuning and validation of the general strategy is surely needed. Work is already in progress along this and related directions.

## Appendix: Properties of Rotational Quaternions

In this section, a brief summary of quaternions and their properties is given; the reader may refer to, e.g., Hanson’s^44^ or other specialized texts for more extensive explanations. Quaternions were originally devised by Hamilton as a generalization of complex numbers, and we used a related notation

20

with the rules

21

22

but they are more commonly represented as an ordered set of four reals or with the so-called angle–axis representation

23

The connection of quaternions to the representation of rotations follows by writing them in terms of Euler angles (θ, ψ, ϕ)

24

25

26

27

Any rotation of a generic three-dimensional (3D) vector **u** about a fixed axis can be represented by the Euler–Rodrigues formula (where **c** is the axis of rotation and ζ is the angle)

28

which can be written in terms of *Q* using relations 24–28 and expanded in matrix form to give the matrix **A** commonly found in textbooks and papers:^a^

29

It can be shown that acting with **A** on **u** is equivalent to eq 1

30

31

meaning that the RVV1 algorithm can be implemented using either rotation matrices or the quaternion operations; here, we used the latter approach for the sake of efficiency (about half of floating point operations are required for a single rotation).

We now examine some properties of quaternion arithmetic that are used in the RVV1 algorithm. A “scalar quaternion” [*q*_0_, **0**] has vector part **q** = [*q*_1_, *q*_2_, *q*_3_] zero, while a “pure quaternion” is vanishing vector part; eliminare ‘zero’ [0, **q**]. Quaternion addition is just

32

and quaternion multiplication can be written as

33

The conjugate of *Q* is defined as

34

and the following relationships can be proven

35

36

37

The norm of *Q* is given by

38

a unit quaternion is such that *N*(*Q*) = *QQ** = 1. Inversion of a quaternion is achieved by

39

hence, *Q*^–1^ = *Q** for unit quaternions. In this manuscript, we consider always unit quaternions for all rotations of reference frames, enforcing normalization every time a quaternion is calculated or updated.

The dependence of *Q̇*(*t*) on *Q*(*t*) (eq 3) is derived as follows. Since we use unit quaternions, *QQ** = 1 is constant, and we can write

from which it follows that *P* = *Q̇Q** has vanishing scalar part and vector part −**q̇** × **q**. Now, we know from eq 1 that **r**(*t*) = *Q*(*t*)**r**(0)*Q**(*t*) and thus **r**(0) = *Q*(*t*)***r**(*t*)*Q*(*t*); taking the time derivatives yields (dropping the dependence of *Q* on *t*)

where ***ω*** is the angular velocity in the laboratory frame. The angular velocity in molecular fixed frame can be calculated as

which gives eq 3.
